# Timing of infections in patients with primary immunodeficiencies treated with intravenous immunoglobulin (IVIg)

**DOI:** 10.1186/s13223-018-0247-8

**Published:** 2018-08-13

**Authors:** Parwinder K. Gill, Stephen D. Betschel

**Affiliations:** 1 Division of Clinical Immunology and Allergy, Western University, St. Joseph’s Hospital, London, ON Canada; 2 Division of Clinical Immunology and Allergy, University of Toronto, St. Michael’s Hospital, Toronto, ON Canada

**Keywords:** Common variable immunodeficiency, Agammaglobulinemia, Immunoglobulin, Infection, IVIg

## Abstract

**Purpose:**

Patients with common variable immune deficiency and X-linked agammaglobulinemia are unable to produce their own antibodies thus leading to a higher incidence of recurrent infections, particularly those involving the sinuses and lungs. Treatment with intravenous immunoglobulin therapy aims to reduce the incidence of infections; however, as serum IgG approaches its trough during the third and fourth week after infusion, we hypothesized that the rate of infection would be higher during this time period.

**Methods:**

Patients with a diagnosis of either common variable immunodeficiency (CVID) or X-linked agammaglobulinemia (XLA) treated with intravenous immunoglobulin (IVIg) were analyzed in a prospective cohort study. Data was obtained as to the timing of symptom onset post infusion, the type of infection, as well as timing of the initiation of antibiotics. Descriptive analyses were conducted to explore the patterns of the data at each month and then over the course of the study year.

**Results:**

Twenty-three patients with a diagnosis of either CVID (n = 22), or XLA (n = 1) were enrolled with a mean follow duration of 11.3 months. The mean number of days to infection after IVIg infusion, the primary endpoint, was 17.0 days with the most common infections reported as sinusitis and upper respiratory tract infections. There was no statistically significant difference (p = 0.70) in the rates of infection when considering the weeks post-infusion.

**Conclusions:**

We believe that this pilot study is the first reported prospective study to examine the timing of infections after IVIg infusion in individuals with CVID and XLA. Further multi-centered research with a larger sample size is required into the comparison of infection rates in primary immunodeficiency patients treated with IVIg versus subcutaneous immunoglobulin therapy, where serum IgG levels remain at steady state.

## Background

Patients with common variable immune deficiency and X-linked agammaglobulinemia are unable to produce their own antibodies. This leads to a higher incidence of recurrent infections particularly involving the sinuses and lungs. To help make up for this lack of antibodies they are treated with IVIg replacement. The aim of this treatment is to reduce the incidence of the above-mentioned infections. Less well studied than the effect of IVIG on infection rates has been the timing of infection relative to the date of receiving IVIG infusion, since serum IgG level wanes over time with each infusion. The purpose of this study was to determine whether or not infections tend to occur in the later weeks after receiving each IVIG infusion. The primary endpoint of this study evaluated the timing and frequency of infections between IVIG infusions.

We hypothesized that the rate of infection for patients receiving monthly IVIG will be higher during the third and fourth week after IVIg infusion, where serum IgG level approaches its trough.

## Methods

### Study design/type

This study was approved by the Research Ethics Board at the University of Toronto. Written informed consent was obtained from all patients before sampling.

After enrolment, patients who required antibiotic treatment for infections were asked to provide the date of onset of their symptoms, as well as the date of first dose of antibiotic. To minimize recall bias, details regarding infection timing were collected at the first and all subsequent IVIg infusions. In addition, sputum cultures and chest X-rays were obtained if appropriate as dictated by standard of care.

Over the duration of the study, monthly IVIg infusions took place and the timing of each infection relative to the preceding infusion was collected. The number of infections was recorded, with emphasis on the onset and treatment of infection (i.e. day 1 to day 28 post infusion).

Subject inclusion criteria were: a diagnosis of CVID (based on the current ESID classification) [1, 2] or XLA; currently receiving or previously received IVIG at 4 week intervals, for at least 6 months; a stable clinical course; over the age of 18; willing to comply with all trial procedures, and sign an Informed Consent Form.

Subjects were excluded if: they had an anaphylactic or severe systemic response to Immune Globulin (Human); were a drug or alcohol abuser; were HIV PCR positive, Hepatitis C PCR positive, or hepatitis B DNA assay positive; had a concomitant disease, the course of which might reasonably be expected to change during the study period; or had other chronic conditions or treatments which, in the investigator’s opinion, may confound the interpretation of the results of the study questionnaires, such as chemotherapy or prophylactic antibiotics. The investigators felt that the inclusion of individuals on prophylactic antibiotics may confound results and lead to under-representation of infections, and in such a small sample size in a rare condition, it may skew results.

A history of past comorbidities and those present at enrollment was obtained through patients’ recollection as well as through clinical documentation.

### Statistical analysis

Descriptive analyses were conducted to explore the patterns of the data at each month and then over the course of the study year. Descriptive statistics were calculated as mean, standard deviation, median, inter-quartile range for continuous variables and proportions for categorical variables.

To evaluate the primary endpoint, the mean number of days to infection among patients who had at least one infection between infusions, we performed a mixed model with random intercepts to account for multiple visits and multiple infections per patient and estimate the mean number of days after infusion.

To estimate infection rates, we categorized days to each new infections since infusion as Week 1 ≤ 7 days, Week 2 8–14 days, Week 3 15–21 days, Week 4 > 21 days. For each patient, we summed up all new infections within each category across all visits. We analyzed the data assuming the Poisson distribution for total repeated counts of new infections at weeks 1, 2, 3, and 4 post-infusion, accounting for within-patient correlations.

Secondary outcomes including the average number of infections per year, types of infections and the use of antibiotics to treated documented infections, were analyzed assuming the Poisson distribution for total counts.

SAS PROC GENMOD was used to fit a generalized estimating equations Poisson model assuming the exchangeable correlation structure and including an offset as the natural log of total years of follow-up. Rates/year within each week post-infusion and 95% confidence intervals were estimated. A time shifted analysis was not conducted in this study.

All analyses were conducted using SAS 9.4 (SAS Inc. Cary, NC). All tests were two-sided and statistical significance was defined if the p value was 0.05 or less.

PROC GENMOD in SAS will be used fit a Poisson model to the data. Patient baseline characteristics such as age, gender, time since diagnosis, and IgG trough levels may be incorporated in all models.

## Results

Twenty-three patients with a diagnosis of either CVID (n = 22), or XLA (n = 1) treated with monthly IVIg in the Division of Clinical Immunology and Allergy at St. Michael’s Hospital, were enrolled in this study. All patients initially satisfied all inclusion and exclusion criteria; however, the mean follow up time in this study was 11.3 months (or 0.9 years), with the mean number of days between visits of 29.6 (range 21–91). The mean age at the time of diagnosis was 32.4 years (range 10–58), with a mean age at enrollment of 46.5 years (range 25–77). Comorbidities related to the primary immunodeficiency, as well as those unrelated, were recorded for each patient (Table 1).

**Table 1:** Past and current comorbidities related to primary immunodeficiency, n (%)

	Past	Current
Pneumonias	18 (78.3)	1 (4.3)
Sinusitis	16 (57.1)	6 (21.4)
Bronchitis	12 (42.9)	4 (14.3)
Bronchiectasis	10 (35.7)	10 (35.7)
Otitis media	6 (21.4)	1 (4.3)
Cytopenias	6 (21.4)	3 (10.7)
Juvenile/rheumatoid arthritis/SLE	2 (7.1)	1 (4.3)
Inflammatory bowel disease	2 (7.1)	2 (7.1)
Autoimmune liver disease	0 (0.0)	0 (0.0)
Granulomatous lymphoid interstitial	0 (0.0)	0 (0.0)
Lung disease (GLILD)		
Celiac disease	0 (0.0)	0 (0.0)

The mean dosage of IVIg per weight at baseline was 565.3 mg/kg. Patients were on one of four treatment products, Gammagard (n = 1), Gammunex (n = 14), Privigen (n = 6) and Octagam (n = 2). The mean trough level at baseline was 10.0 g/L (Table 2). The patients’ IVIg treatment was continued at the same dose and frequency as it was prior to entering the study, unless changes were required for medical reasons independent of this study.

**Table 2:** Demographics and baseline characteristics (n = 23 unless otherwise indicated)

Gender, n (%)	
Female	13 (56.5)
Male	10 (43.5)
Age at diagnosis (years) [n = 21]	
Mean (range)	32.4 (10–58)
Age at baseline (years) [n = 23]	
Mean (range)	46.5 (25–77)
Weight at baseline (kg)	
Mean (range)	70.5 (50.3-94.9)
Immunoglobulin trough level at baseline (g/L)	
IgG mean (range)	10.0 (3.3–13.1)
IgA mean (range)	0.19 (0.07–1.13)
IgM mean (range)	0.18 (0.04–1.17)
Dosage per weight at baseline (mg/kg)	
Mean (range)	565.3 (441.0–714.3)
Treatment product, n (%)	
Gammagard^®^	1 (4.4)
Gamunex^®^	14 (60.9)
Privigen^®^	6 (26.1)
Octagam^®^	2 (8.7)
Primary immunodeficiency disease, n (%)	
Common variable immunodeficiency	22 (95.7)
X-linked agammaglobulinemia	1 (4.3)

Two patients were withdrawn due to length of time between follow up visits, one due to a newly diagnosed malignancy, and one patient was withdrawn as they were started on prophylactic antibiotics during the course of the study. Their data were included in the statistical analysis up until the point of withdrawal. Two patients required dosage increases of IVIg during the course of the study, one due to weight-base changes, and the second due to low immunoglobulin levels despite replacement. This highlights the possibility of the ineffective of their immunoglobulin replacement therapy.

The overall number of infections across all patients and all visits was 63 reported in 258 visits; of these, 56 were reported as new infections at the subsequent follow up visit. Seven infections were excluded from analyses as five were documented as infections persisting from the last visit, and two infections had unknown dates. The 56 new infections were accounted for in 19 patients, with no reported infections in the remaining four individuals. For individuals who required dose adjustments during the study, no new infections were reported in these few patients. Furthermore, out of all infections documented, only three new infections were reported in individuals receiving IVIg within 21 days of the last infusion versus the remaining 53 infections which occurred more than 21 days since the last infusion.

The primary endpoint of the study, to evaluate the mean number of days to infection post-infusion amongst patients who had at least one new infection between infusions, was 17.0 with a 95% confidence interval of 14.5–19.5.

Secondary outcome measures in this study included the rate of infections, types of infections, as well as the treatment of each infection. Overall, in 258 visits, the proportion of new infections across all visits was 21.7%. The rate of new infections per patient year was 2.64, stratified by week post-infusion was calculated as follows (Table 3): During the first week post-infusion, the rate of new infections per year as 0.52, during the second week 0.62, the third week 0.71, and the rate during the fourth week was 0.81. Although slightly higher values were found in the third and fourth weeks post-infusion, there were no statistically significant differences in post-infusion infection rates across all weeks (p = 0.7001).

**Table 3:** Frequency of infections

Type of infection	Frequency	Percent (%)
Sinus	31	49.21
Upper respiratory tract	21	33.33
Eye	4	6.35
Ear	3	4.76
Other^a^	4	6.35

^a^Diarrhea, Cutaneous infections

The most common illnesses experienced out of a total 63 reported were sinus infections in 49.2% of cases (n = 31), upper respiratory tract infections in 33.3% (n = 21), followed by eye infections in 6.35% (n = 4), ear infections in 4.76% (n = 3), with 6.35% (n = 4) cases represented by GI and cutaneous infections. In 5 of these 63 cases, patients reported their symptoms persisting until the next follow up visit despite treatment with antibiotics.

Of these 63 cases, 12 infections were left untreated, with 1 unknown antibiotic. The most common antibiotics prescribed were as follows: Clarithromycin (n = 13), Amoxicillin (n = 11), and Levofloxacin (n = 6).

## Discussion

Individuals with primary immunodeficiencies often experience recurrent infections as a manifestation of their underlying disease, most often due to deficient or absent antibody production. The goal of immunoglobulin replacement therapy is to minimize the rate and occurrence of severe infections and hospitalizations and improve overall quality of life [3]. The optimal route of administration, whether intravenous or subcutaneous, has not been established and is dependent on a number of variables including patient characteristics and preferences. Anecdotally, the administration of IVIg has been associated with a ‘wear off’ effect and the risk of increased susceptibility to infections as the IgG level approaches its trough at the end of a typical infusion cycle of 4 weeks.

To our knowledge, this pilot study is the first published prospective study to examine the timing of infections post-infusion in individuals with primary immunodeficiencies treated with IVIg. We hypothesized that as IgG levels approach their trough values in the third and fourth weeks after infusion, the infection rates would be higher due to a ‘wear off’ effect. Although there were no statistically significant differences in the rates of infection per year stratified per week, the mean number of days to infection was 17.0, falling within the third week after infusion. The lack of statistical significance could be explained by the small sample size of the study, which could be addressed by larger studies going forward. Given that our results did not meet statistical significance, we cannot conclude whether this in fact supports our hypothesis; however, the majority of infections recorded (53/56) occurred more than 21 days after the patient’s infusion. Going forward, multi-centre research with a larger sample size may provide more insight.

For the purposes of this study, individuals using prophylactic antibiotic were excluded as the investigators felt that this may in fact skew results. As reported in Table 2, the trough levels of our patient population were variable, ranging from 3.3 to 13.1 g/L (mean = 10 g/L); Patients were included irrespective of baseline trough levels, given the notion of targeting an individuals’ ‘biologic’ trough level rather than a set value. To note, there was only one outlier with a value of 3.3 g/L while the median trough level at baseline was 10 g/L. Stratification of infection timing in the context of baseline trough levels was not conducted. Furthermore, although consideration could be given to the increased number of infections in the winter months, given our small patient population, further stratification, and time shifted analysis were not conducted.

This ‘wear off’ effect has been investigated in prior research conducted by Zbrozek et al. [4] examining infections in the context of healthcare utilization in clinics, emergency rooms and hospitalizations. For the purpose of this study, the ‘wear-off’ effect was used to refer to objectively determined infections, rather than individuals’ subjective reports of symptoms that may be related to infusions such as fatigue and malaise, as these would be difficult to objectively define. A greater number of infections was noted in the time period greater than 18 days after infusion, with higher rates of health care utilization over a period of seven months [4].

A pooled analysis conducted by Bexon et al. [5] evaluated the timing of infections in relation to immunoglobulin administration, showing that the highest rate of infections was noted in the last week of a 3 and 4 week dosing cycle [4]. Less than 8% of the visits (20/258) occurred 21 days between infusions, and of the new infections reported, only 3 out of 5 occurred during visits that were 21 days apart. Yearly infection rates have previously been evaluated in patients receiving both intravenous and subcutaneous forms of immunoglobulin replacement. Borte et al. [6] and Suez et al. [7] reported rates of 4.38 and 2.41 infections per patient year respectively, in those receiving subcutaneous immunoglobulin, and Wasserman et al. [8] reported a yearly rate of infections similar to the rate observed in our study (2.64 infections/patient year) at 2.6 infections per patient year. This highlights the importance of determining not only the optimal method of replacement, but also ensuring the efficacy.

Challenges of such studies include patient recall bias of the onset and timing of symptoms of infection and the initiation of antibiotics due to data collection methods. Patients were not required to keep a diary of infections as they were seen at most every 4 weeks; rather, at each subsequent visit or by telephone, they were asked to recall details regarding any inter-current illnesses as well as the use of antibiotics. As noted by Bexon et al. [4], there may also be a carry-over effect where infections reported shortly after infusion may have had symptom onset in the days leading up to the infusion.

Another limitation of the study would be the small sample size given our patient population; this may have affected the overall statistical significance; thus, it would be useful to conduct such a study on a larger scale with a broader patient population.

Given the concerns of this ‘wear off’ effect and the association with an increased number of infections as the IgG level reaches its trough, further investigation is needed to compare infection rates and the timing of infections in patients treated with intravenous versus subcutaneous immunoglobulin therapy. As previously mentioned, several patients with withdrawn from the study due to the development of a malignancy, the use of prophylactic antibiotics, and persistently low immunoglobulin levels. The results of this study hold larger implications for patient care, as if a true ‘wear-off’ effect with respect to infections can be ascertained, this may necessitate adjustments in both the timing and dose of therapy, as well as consideration of alternative routes of administration, such as subcutaneous therapy. As these patients are at risk of recurrent and severe infections, the optimal dosing and administration of immunoglobulin therapy is of utmost importance in minimizing these risks and improving overall quality of life [9].
